# Alginate encapsulated multipotent adult progenitor cells promote corneal stromal cell activation via release of soluble factors

**DOI:** 10.1371/journal.pone.0202118

**Published:** 2018-09-07

**Authors:** Olla Al-Jaibaji, Stephen Swioklo, Kristel Gijbels, Bart Vaes, Francisco C. Figueiredo, Che J. Connon

**Affiliations:** 1 Institute of Genetic Medicine, Newcastle University, Newcastle upon Tyne, United Kingdom; 2 ReGenesys BVBA, Heverlee, Belgium; 3 Department of Ophthalmology, Royal Victoria Infirmary, Newcastle upon Tyne, United Kingdom; Cedars-Sinai Medical Center, UNITED STATES

## Abstract

To reduce the increasing need for corneal transplantation, attempts are currently aiming to restore corneal clarity, one potent source of cells are multipotent adult progenitor cells (MAPC^®^). These cells release a powerful cocktail of paracrine factors that can guide wound healing and tissue regeneration. However, their role in corneal regeneration has been overlooked. Thus, we sought to explore the potential of combining the cytoprotective storage feature of alginate, with MAPC to generate a storable cell-laden gel for corneal wound healing. 72 hours following hypothermic storage, alginate encapsulation was shown to maintain MAPC viability at either 4 or 15°C. Encapsulated MAPC (2 x10^6^ cells/mL) stored at 15°C presented the optimum temperature that allowed for cell recovery. These cells had the ability to reattach to tissue culture plastic whilst exhibiting normal phenotype and this was maintained in serum-free and xenobiotic-free medium. Furthermore, corneal stromal cells presented a significant decrease in scratch-wounds in the presence of alginate encapsulated MAPC compared to a no-cell control (p = 0.018). This study shows that immobilization of MAPC within an alginate hydrogel does not hinder their ability to affect a secondary cell population via soluble factors and that these effects are successfully retained following hypothermic storage.

## Introduction

Corneal damage and opacity have been estimated to cause blindness in 8 million people (c.10% of total blindness) worldwide each year [1]. Corneal stroma constitutes 90% of the corneal structure containing keratocytes, collagen fibrils, and proteoglycans, all of which help maintain vision [2]. Corneal keratocytes usually remain quiescent and are only activated when penetrating damage to the tissue occurs [3]. Usually, a sequence of complex biological events work together to promote corneal wound healing, including cell migration, proliferation, extracellular molecule (ECM) disposition and secretion of angiogenesis factors. While corneal transplantation is the most utilised surgical intervention for treating corneal damage, it still has significant limitations such as corneal availability and compatibility. Cell therapy is a promising technique that has shown vast potential, evidenced by an escalating number of reported cell therapies around the world. Cell therapy has been investigated for the treatment of a range of diseases; such as, heart disease, neurodegenerative disorders, cancer, limb ischemia, and loss of sight, among many others. So far, very few cell-based therapy products have been approved by the European Medicines Agency and the Food and Drug Administration (FDA) [4–8].

Multipotent adult progenitor cells (MAPC^®^) present a promising source of therapeutic cells. MAPC are derived from a primitive cell population that can be harvested from bone marrow, muscle and brain [9]. MAPC are a more primitive cell population than mesenchymal stem cells (MSCs), whilst they imitate embryonic stem cells characteristics they still retain adult stem cells potential in cell therapy. *In vitro*, MAPC demonstrated a vast differentiation potential to adipogenic, osteogenic, neurogenic, hepatogenic, hematopoietic, myogenic, chondrogenic, epithelial, and endothelial lineages. A key feature of MAPC is that they show large proliferative potential in vitro without losing their phenotype [10]. The therapeutic potential of MAPC has been documented in many diseases such as ischaemic stroke [11], graft versus host disease [12], acute myocardial infarct [13], organ transplant [14] bone repair and myelodysplasia [15–17]. MAPC have also been shown to enhance bone formation, promote neovascularisation, and have immunomodulatory effects [18, 19]. Therefore, they may have significance for corneal wound healing as indicated in previous studies, including the documented potential for MSC-derived immunomodulatory growth factors such as tumor necrosis factor-inducible gene 6 protein (TSG-6) to improve corneal transparency following wounding [20].

There are many important considerations for the successful implementation of cell-based therapy including how the cells are delivered, but also maintained at the site of repair. Cell delivery would include cell transportation from the laboratory to the treatment site and direct application of the cells to the patient. Hydrogels offer a solution to both of these processes as they are capable of retaining cells within a highly hydrated environment whilst being strong enough to resist immediate damage in a physiological environment and subsequent release of the cells during application on the patient. Thus, encapsulation of therapeutic cells within a hydrogel offer improvement in storage, transportation and delivery. Hydrogels formed from sodium alginates are found in nature and are readily extracted from brown seaweed [21]. Alginate hydrogels have excellent safety and toxicity records, as such, they have been used widely in therapy [22], wound dressings [23], but also in protein and cell immobilization [24, 25]. The relative simplicity and cell-friendly nature of alginate hydrogel gelation have strengthened its position as a leading biomaterial in cell therapy [26]. Recently alginate encapsulation has been shown to offer protection to MSCs when exposed to hypothermic temperatures for prolonged periods (days to weeks) [23, 24]. It was also shown that MSCs had the optimum viable recovery at 15°C when encapsulated in alginate [27]. Therefore, alginate provides a simple, low-cost alternative to cryopreservation for short-term storage and transportation including both cell retrieval to manufacturing site and subsequent delivery to the treatment center. Furthermore, such an approach may offer greater flexibility in patient scheduling and transportation. Interestingly, the ability for alginate hydrogels to retain encapsulated cells inside the body in a functional state, could work in combination with its ability to preserve cells during hypothermic storage to create a storable cell-laden gel. Such a medical device could be manufactured centrally, then shipped and stored at hypothermic temperatures before being applied as hydrogel bandage containing therapeutic cells to directly affect a superficial wound. Unlike traditional approaches to cell therapy, the cells could remain encapsulated within the bandage and induce wound healing response via the release of paracrine growth factors. Thus, within this study, we investigated the potential of alginate-encapsulation of MAPC to maintain cell viability, morphology, and ability to affect the closure of an *in vitro* corneal stromal scratch-wound via paracrine factors following 72 hours of hypothermic storage at 4 and 15°C.

## Materials and methods

### Ethics

Corneal tissues were obtained as by-products of grafting procedures, and kindly provided by Dr Franscisco Figueiredo, MD FRCOphth, Royal Victoria Infirmary Newcastle, UK, following informed consent in accordance with Newcastle University and Newcastle-upon-Tyne Hospital Trust Research Ethics Committees guidelines. Human multipotent adult progenitor cells (MAPC) were obtained in collaboration with ReGenesys, Belgium. MAPC were obtained with consent from a healthy donor.

### Human corneal stromal cells isolation and expansion

Human corneal stromal cells were extracted from the excised corneal rings of healthy human cadaveric donors at the time of corneal transplantation, Corneal tissues were minced using scalpel after debriding epithelial and endothelial cells. Stromal cells were extracted via enzymatic digestion using Dulbecco’s Modified Eagle Medium (DMEM/F12) (ThermoFisher Scientific, Loughborough, UK) supplemented with 5% fetal bovine serum (FBS), 1% penicillin-streptomycin (ThermoFisher Scientific) and 2 g/L collagenase type I (Sigma-Aldrich, UK). Tissues were then incubated in a humidified incubator (37°C, 5% CO_2_) for 5 hours under rotation. The cells were subsequently dissociated with 0.25% Trypsin-EDTA (ThermoFisher Scientific) solution for 10 min and filtered through a 40 μm EASYstrainer^™^ (Greiner Bio-One, UK). Lastly, the solution was neutralized using the DMEM/F12, and centrifuged at 1500 xg for 5 min followed by re-suspension and seeding in a tissue culture flask (ThermoFisher Scientific) with DMEM/F12 and immediately placed inside the incubator with media change every two days. At 80% confluence, cells were dissociated using TrypLE^™^ express enzyme (ThermoFisher Scientific) and expanded for the experiments, and cells were used up to passage 4. For the start of the experiment, corneal stromal cells were plated in a 6-well plate at a density of 2 x 10^5^ cells per well with DMEM/F12 for one day. Followed by a medium change to serum-free DMEM/F12 supplemented with 1 x 10^−3^ M L-Ascorbic acid (Sigma-Aldrich), Insulin-Transferrin-Selenium (ITS-G) 1:100 (Invitrogen) and penicillin/streptomycin 1% (SFM).

### MAPC collection and isolation

MAPC were obtained in collaboration with ReGenesys, Belgium. Briefly, the cells were isolated according to previously described methods [28] from a single bone marrow aspirate. MAPC were expanded and maintained in xenobiotic-free growth medium (XF MAPC medium, ReGenesys, Heverlee, Belgium). Our cells expressed characteristic morphological features of MAPC as described previously by Crabbé et al [29] and stored in liquid nitrogen until required. The cells, once thawed, were seeded at 2 x 10^3^ cells per cm^2^ on CellBIND tissue culture plastic (Sigma-Aldrich) and maintained in XF MAPC medium in a humidified incubator. Once cells had reached 80% confluence, they were detached using TrypLE Express Enzyme to harvest the cells prior to encapsulation in alginate. Cell number was assessed by incubating them with 1 μM Calcein-AM (CAM) and 2 μM Ethidium Homodimer-1 (EthD-1) (Thermo Fisher Scientific) for 15 minutes before counting viable (CAM+) and dead (EthD-1+) cells using a Countess II FL automated cell counter (Invitrogen).

### MAPC encapsulation and storage

**Fig 1:**
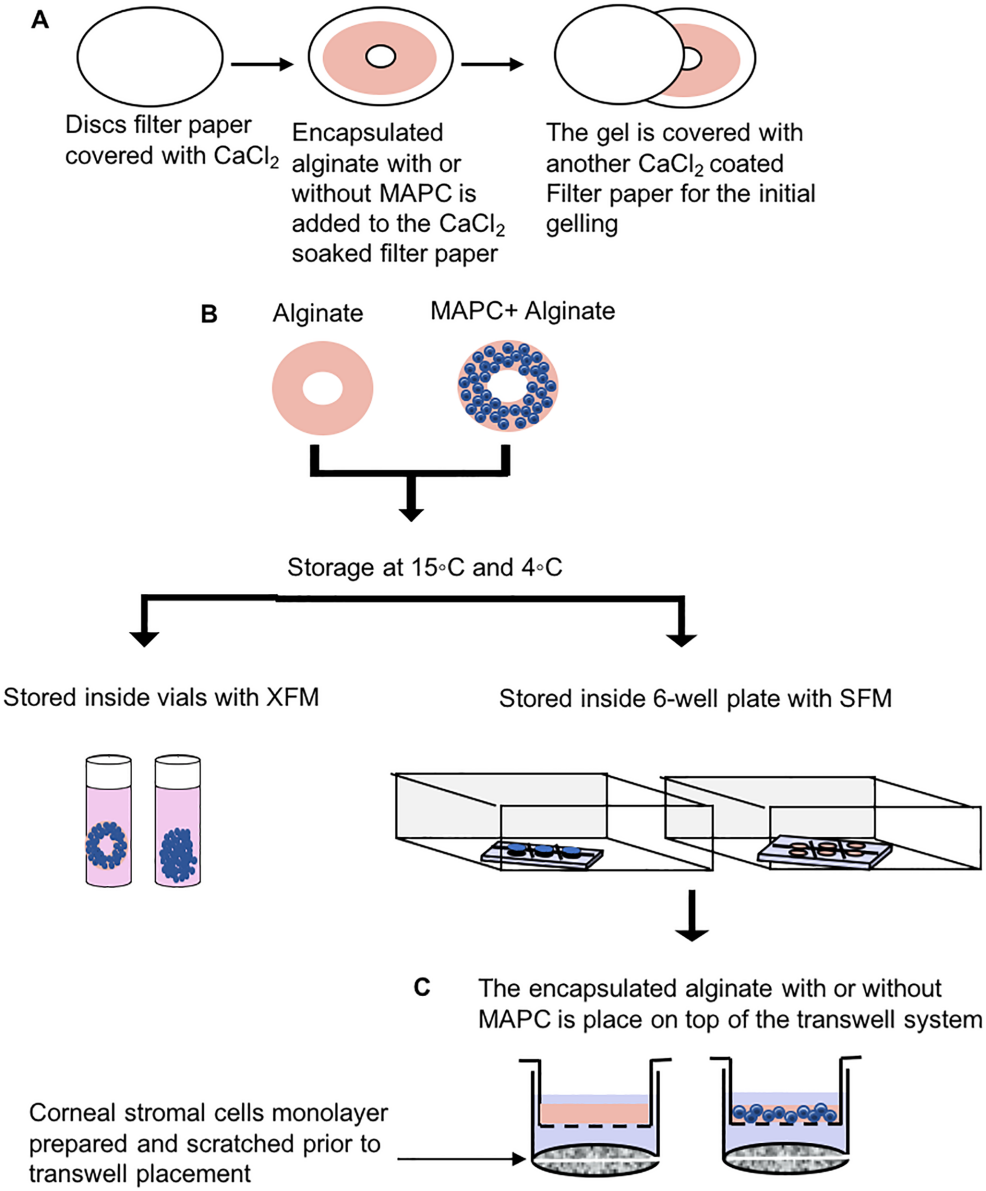
A schematic representation of the encapsulation and storage process of alginate with or without MAPC. (A) The initial gelling of the alginate prepared by transferring the mixed alginate with or without MAPC into the CaCl_2_ masked filter paper and left for two minutes before allocating the gel into 4 ml CaCl_2_ for final gelling. (B) The formed encapsulations are either stored in a cryovials or in an air tight chamber for 72 hours at (4 and 15°C). (C) Following corneal stromal cell scratch-wound induction, the gel is situated on the upper compartment of the transwell to avoid direct contact between the gel and the corneal stromal cells monolayer. MAPC, Multipotent adult progenitor cells; SFM, serum-free DMEM:F12 medium; XFM, xenobiotic-free MAPC medium.

A solution of 0.5 ml 2.4% (wt/vol) sodium alginate (NovaMatrix, FMC, Norway) containing a suspension of 1 x 10^6^ cells was prepared in SFM or XFM. This solution was then gelled by crosslinking the alginate using an excess of 102 mM calcium chloride within disc-shaped molds (thickness 1mm, diameter 24mm) as previously described [30] (Fig 1A). The formed discs containing encapsulated MAPC were washed briefly with sterile phosphate buffered saline (PBS) (Sigma-Aldrich) and then transferred to either 2ml cryovials (with cell only controls) with 1 ml XFM or to a transparent Transwell with 0.4 μm pore size (Greiner Bio-One LTD, UK) (with gel only controls) with SFM within a 6 -well plate and sealed (by an airtight cap or parafilm respectively) and kept within a polystyrene box placed in a cooled incubator set to either 4°C or 15°C (Fig 1B). The gelled discs for subsequent experiments were immediately used following 72 hours of hypothermic storage at 4°C and 15°C. Following hypothermic storage, the gels containing cells were briefly allowed to equilibrate to room temperature before further use. Gel only controls (i.e. no cells) were treated in the same conditions as described above.

### Cell release and viability assessment

The encapsulated MAPC were released for viability assessment. Cells were released by dissolving the alginate gel using 3 mL 100 mM trisodium citrate (Sigma-Aldrich) for 5 minutes at room temperature. Cell suspension was further diluted with 7 ml PBS before separating cells by centrifugation and dissociating them with 0.2 ml TrypLE^™^ Express enzyme for 5 minutes (due to aggregation of cells at this stage in the control, non-encapsulated samples). An equivalent volume (0.2 ml) of media was then added, and cells were carefully re-suspended prior to counting. The number of viable cells released was enumerated by incubating cells with 1 μM Calcein-AM and 2 μM EthD-1 for 15 minutes at 37°C before counting. The cell only control samples were treated using the same method as the encapsulated cells.

### MAPC attachment and functional cell recovery assessment

Following 72 hours storage, the released MAPC were counted and plated at 2 x 10^4^ cells/cm^2^ in a CellBIND (Sigma-Aldrich) 24-well plate and incubated for 24 hours in a humidified incubator (37°C, 5% CO_2_) before capturing images. Attached cell number was assessed using the alamar blue assay. After 1 hour of incubation at 37°C, fluorescence was taken at an excitation of 545 nm and emission of 590 nm using Varioskan^™^ LUX Multimode Microplate Reader (ThermoFisher Scientific). Plates were then stained with methylene blue to assess cell number as previously described [31]. After washing with sterile PBS, absorption was measured at 650 nm using Infinite F50 plate reader (Tecan Life Sciences, UK). Cell number was assessed using a standard curve produced from non-stored cells. Attachment efficiency was calculated as the number of attached cells divided by the number of seeded cells. Functional cell recovery was calculated as the number of viable cells multiplied by the attachment efficiency.

### Scratch-wound assay

Monolayers of human corneal stromal cells were grown to 90% confluence in a 6-well plate, serum-starved for three days and briefly washed with PBS prior to scratching using 1 ml micropipette tip. The scratch-wound was followed by a wash twice with PBS to remove any cellular debris. The scratched cell monolayer was then co-cultured in the presence of encapsulated MAPC after storage at 4°C and 15°C, for 40 hours (Fig 1C). The Transwell membrane prevents direct contact by physically separating the scratched cell monolayer and the alginate hydrogel containing the MAPC. The rate of wound (scratch) closure was quantified using a holographic microscope, Iprasense Cytonote (IPRASENSE, Clapiers, France) that employs lensless imaging technology for real-time monitoring of the cells. Images collected at different time points were processed using ImageJ 1.50e software. The wound areas were manually measured using Polygon selection tool and analyzed using measure plugin. Data were collected from at least three independent biological donors and two technical repeats for each donor.

### Assessment of collagen production

Following wound healing, corneal stromal cells were gently washed with warm PBS before fixing with 70% ice-cold ethanol for 10 minutes and incubated overnight with Picro-Sirius Red stain (Sigma-Aldrich) as previously described [32] with gentle agitation to allow for equilibrium staining of the cells. Afterwards, the plates were thoroughly washed with deionized water to remove the excess dye before adding 1M sodium hydroxide (Sigma-Aldrich) with gentle agitation. Absorbance was measured at 490 nm to measure total collagen amount. Data were normalized to cell number measured using Alamar blue taken at the same time point, fluorescence taken at 540–580 nm. Collagen amount was calculated as the total collagen produced divided by the final cell number.

### Statistical analysis

Statistical Analysis was carried out using GraphPad Prism (V6.00) software (GraphPad Software, Inc, California, USA). Data are conveyed as the mean of values from at least three distinct donors ± SEM. Comparisons of statistical values were analyzed using two-way repeated measures of variance ANOVA, and Tukey’s multiple comparisons tests p-values < 0.05 were considered significant (*, p < 0.05; **, p < 0.01; ***, p< 0.001).

## Results

### Xenobiotic-free medium promotes morphology and survival of MAPC

**Fig 2:**
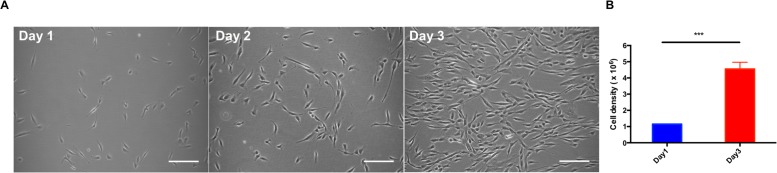
MAPC morphology. (A). Following 3 days’ expansion the cells presented normal spindle-shaped fibroblastic-like cells. Cells had an increase in their proliferation evident by the increase in cell number compared to day1 (B). Scale bar = 200 μm. Values are expressed as means ± SEM from 3 separate experiments with unpaired t test (***p < 0.001).

Starting with exploring the effect of XFM media on MAPC cultures phenotype, MAPC thawed from liquid nitrogen seeded in a 2000 cells/cm^2^ density and grown and maintained in XFM medium for 72 hours at 37°C were analyzed for morphology, proliferation, and viability. Phase-contrast micrographs showed that cells acquired their typical small, spindle-shaped, fibroblastic-like morphology [33, 34]. This shape persisted over three days in culture as was previously shown [28] (Fig 2A). In regards to proliferation, at day three, there was an increase in live cell number compared to the starting seeding density (Fig 2B). Cells viability were analyzed by live/dead double staining at day three, where it showed an average viability of 95 ± 1% of cells.

### Hypothermic storage preserve encapsulated MAPC viability and cell recovery

**Fig 3:**
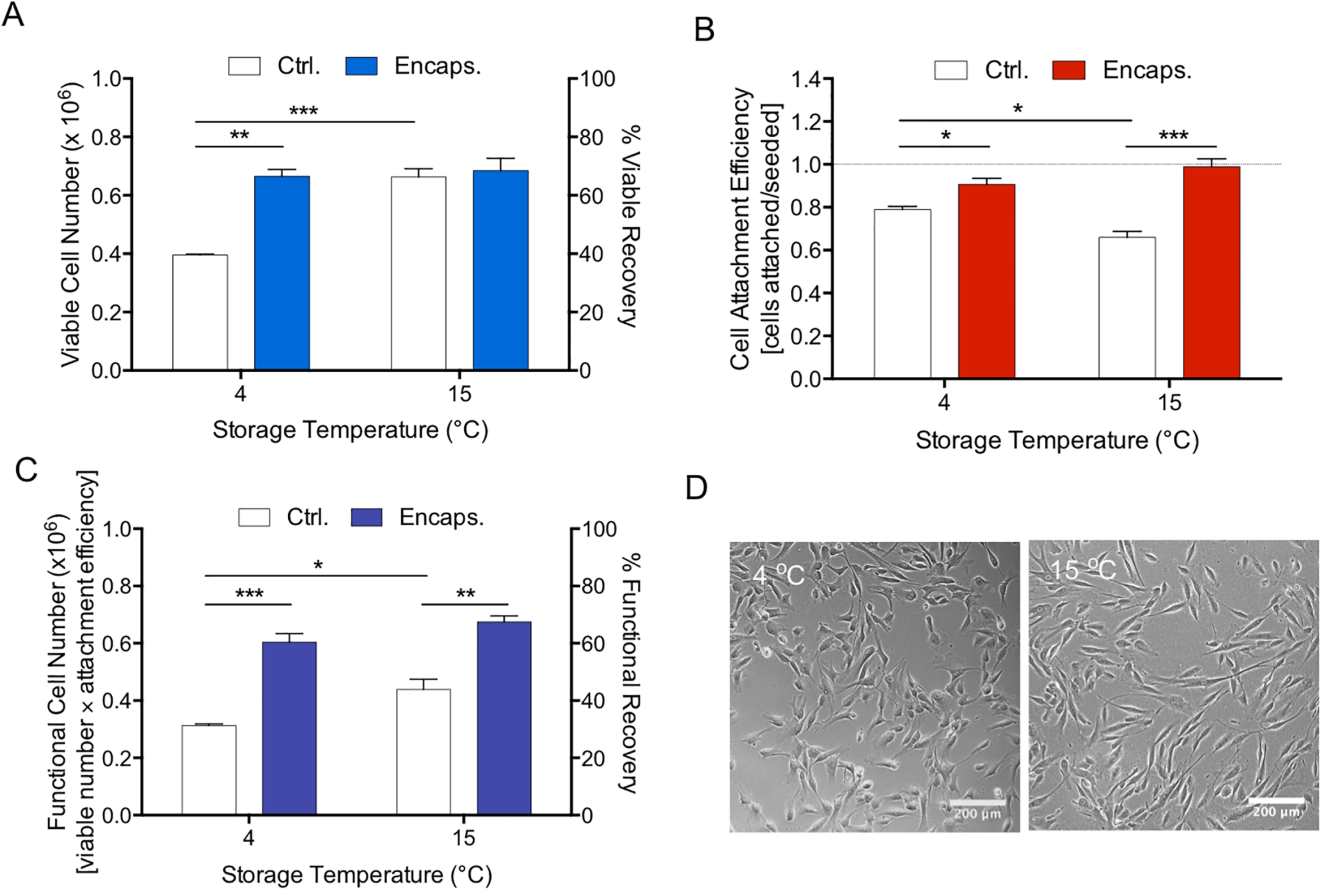
The effect of alginate-encapsulation on the hypothermic preservation of MAPC. MAPC were stored at 4 or 15°C temperatures either encapsulated (Encaps.) or non-encapsulated (Ctrl.) for 72 hours before assessing viable cell recovery (A). Following return to cell culture environment for 24 hours, cell attachment efficiency (B) was assessed. Functional cell number (C) was calculated as the viable cell recovery multiplied by the attachment efficiency. Cell morphology (D) was retained following release from encapsulation and plated for an additional 24hrs under normal conditions. Scale bar = 200 μm. Values are expressed as means ± SEM from 3 separate experiments with asterisks representing significance from control values (***p < 0.001; **p < 0.01; *p < 0.05).

In order to quantify the effect of hypothermic storage on cell viability, we encapsulated 1 x 10^6^ MAPC in 1.2% alginate discs and stored them at 4 or 15°C for 72 hours with XFM medium. Viable cell recovery of encapsulated MAPC were compared with non-encapsulated (controls) MAPC samples. Alginate encapsulated and stored MAPC at both 4°C and 15°C yielded a similar viable cell recovery (approximately 66%) whilst viable cell recovery from control (non-encapsulated) at 4°C was significantly lower (approximately 40%). Control cells stored at 15°C showed no significant difference compared to encapsulated MAPC in viable cell recovery immediately following release (Fig 3A). However, following reattachment significant differences were seen between controls and encapsulated at both 4 and 15°C, suggesting that a considerable number of cells that were shown to be viable by Calcein AM staining, immediately following release were actually not able to reattach, proving functionality had been preserved by alginate encapsulation (Fig 3B). A further measure of cell functionality was attempted by multiplying the values of cell viability by cell attachment efficiency (Fig 3C). This clearly showed a significant protective effect given by alginate encapsulation for hypothermic storage of MAPC, with 15°C offering a slightly higher value than 4°C for encapsulated cells and a significantly greater effect between 4 and 15°C controls. After 24 hours in culture encapsulated cells stored at both 4 and 15°C had attached readily to tissue culture plastic and adopted a normal spindle shaped fibroblast-like morphology indistinguishable from non-stored cells (Fig 3D).

### Encapsulated MAPC promote scratch-wound closure

**Fig 4:**
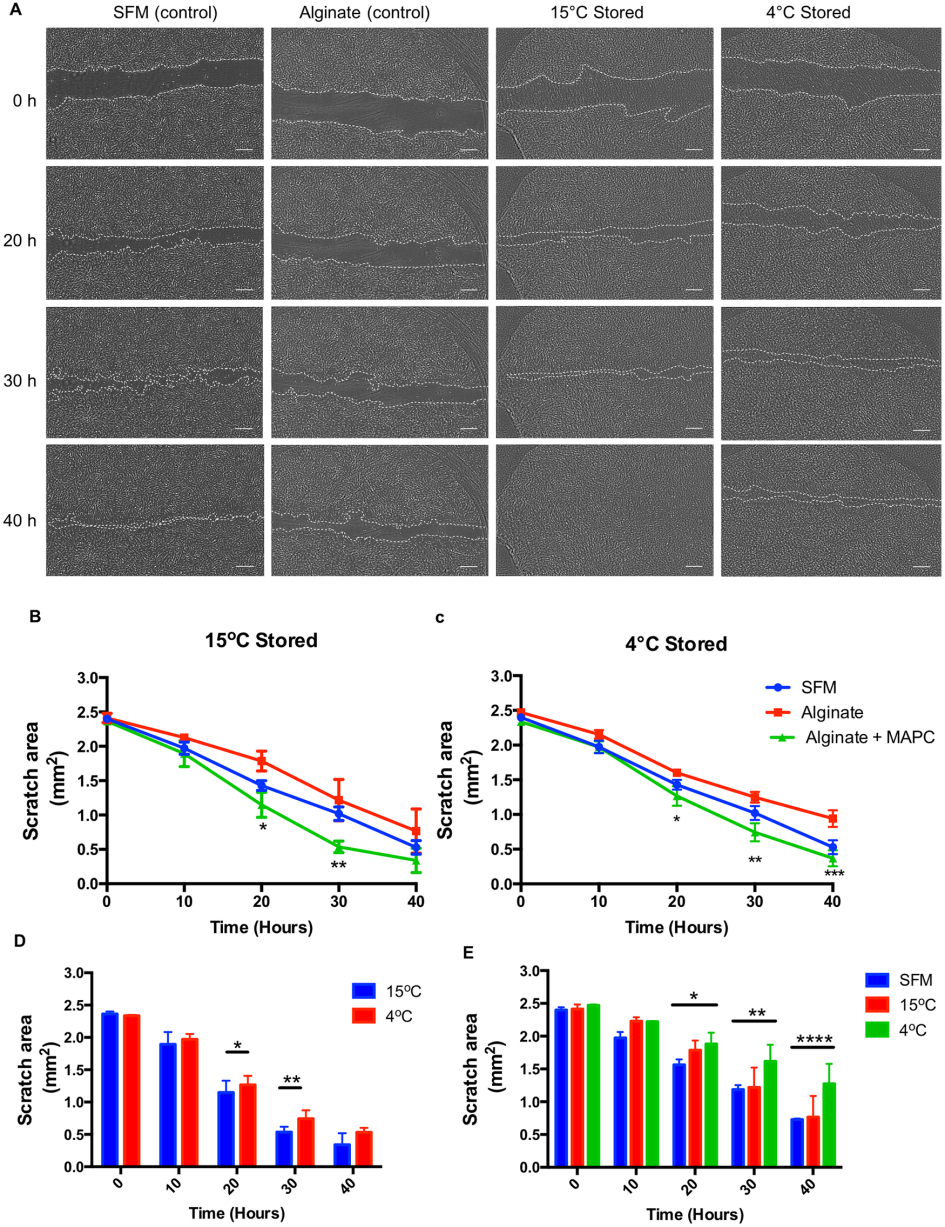
The effect of encapsulated MAPC on corneal wound healing. (A) Representative images using time-lapse microscopy of the scratch-wound of corneal stromal cells treated with serum-free medium (SFM), alginate, or 1 x 10^6^ MAPC alginate + MAPC at time points 0, 20, 30 and 40 hours. Treatments were stored for 72 hours at 15°C (B) or 4°C (C), p-value for the comparison between alginate and alginate + MAPC. Comparison between the encapsulated MAPC at different storage temperatures (D), and alginate only controls at different temperatures compared to SFM control (E). Values are presented as mean ± SEM from 3 separate experiments with asterisks representing significance between values (****, p <.0001; ***, p <.001; **, p <.01; *, p <.05). Scale bars = 300 μm.

**Fig 5:**
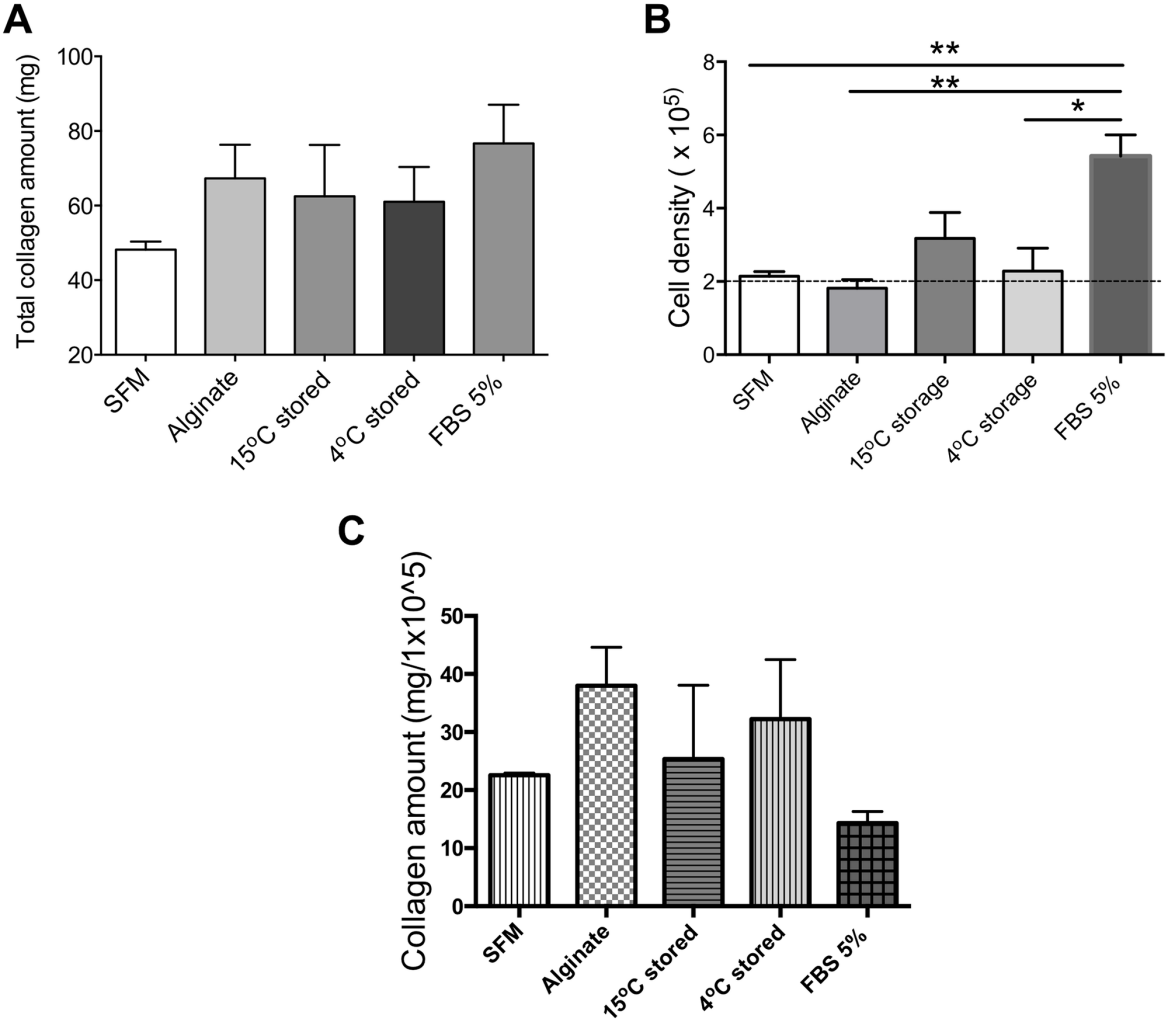
Collagen amount produced 72 hours post-scratch in corneal stromal cell cultures. (A) Total collagen amount measured using Picro-sirius red staining. (B) Cell number measured using Alamar blue assay. (C) Collagen amount normalized to cells number. Values are presented as the mean ± SEM of three different human corneal stromal cell donors.

The effect of encapsulated MAPC on corneal wound healing was examined using a standard scratch-wound assay (Fig 4). Accordingly, the serum-starved corneal stromal cells, as a confluent monolayer, were scratched and incubated in SFM in the presence of encapsulated MAPC (previously stored 4 and 15°C) for two days under normal culture conditions. Immediately following the scratch, cells began migrating to the cell-free area. Wound area closure of human corneal stromal cells was effectively improved in the presence of encapsulated-MAPC when compared to the controls (Fig 5A). At 20 hours, post-scratch, the cultures treated with encapsulated MAPC previously stored for 3 days at 15°C, exhibited a significant increase in wound closure compared to the alginate only control (p = 0.0018) (Fig 5B). Similarly, encapsulated MAPC previously stored at 4°C also significantly increased wound closure compared to alginate controls at 20 hours (p = 0.0002) (Fig 5C). Encapsulated MAPC at 15°C showed similar improvement in wound closure compared to 4°C storage (Fig 5D). Conversely, the presence of alginate only control in scratch-wound culture appears to attenuate the rate of wound healing compared to medium only (SFM) controls (Fig 5E).

### Collagen production

Next, we measured collagen deposition 72 hours post-scratch (at which point all scratches were 100% confluent). Using Picro-sirius red staining we analyzed collagen production from corneal stromal cells (Fig 5A). 72 hours post-scratch, the total collagen amounts were found to be 48.2 mg ± 3.0 in SFM control, whereas increased amounts were found in cultures supplemented with 5% FBS (control) 76.6 ± 14.6, whilst cells co-cultured with encapsulated MAPC were also found to have increased collagen production (62.5 mg ± 19.4 and 61.0 mg ± 13.1 when exposed to 15°C and 4°C stored encapsulated MAPC respectively). Interestingly the alginate alone (gel only control) also showed a similar increase 67.2 mg ± 12.7. However, no significant differences were found among the treatment groups. Cell number was also measured using Alamar blue assay after 72 hours of initial scratching (Fig 5B). Compared to the initial seeding density of 2x 10^5^ cells, it was found that corneal stromal cell number increased slightly to 2.1x 10^5^ ± 0.17 in SFM, decreased slightly to 1.8 x 10^5^ ± 0.32 in presence of alginate controls, and increased to 3.1 x 10^5^ ± 0.1 and 2.2 x 10^5^ ± 0.88 in the presence of 15 and 4°C stored MAPC respectively. As expected adding serum to the media increased corneal stromal cell number considerably (5.4 x 10^5^ cells ± 0.81). Due to the differences in cell number a normalised measure of collagen disposition was taken by dividing the total collagen amount by the total final cell number to measure collagen production per 1 x 10^5^ cells (Fig 5C).

## Discussion

MAPC have previously been shown to have promising therapeutic immunoregulatory effects driven ostensibly by their ability to produce paracrine effector molecules [35]. As such the secretory behavior of these cells has successfully shown to be influential in the treatment of a variety of clinical conditions, such as hind limb ischemia [36], multiple sclerosis [37], myelodysplastic syndrome [16], acute ischemic stroke [11], and bone repair [33]. However, unlike MSCs, MAPC have not yet been tested *in vitro* on corneal stromal cell growth. MAPC have shown many similarities to MSCs [38]. A number of recent papers have described the paracrine effect of MSCs on a variety of corneal wounds *in vitro* and *in vivo* [39, 40]. An important aspect of MSCs is their anti-inflammatory effects, which could inhibit inflammation in the damaged tissue [41, 42]. This was demonstrated in numerous animal models following corneal chemical burn. For example, MSCs-treated rat corneas were found to reduce the levels of matrix metalloproteinase-2 (MMP-2), interleukin-2 (IL-2) [43] and interferon-y (IFN-y) [20]. Another key factor found to reduce inflammation in a mouse corneal wound model is TSG-6 [44]. It has also been reported that MSCs can restore corneal transparency and reduce neovascularization following wounding in a rabbit corneal model, where MSCs were found to reduce corneal opacity and neovascularization via reduction in matrix metalloproteinase-9 (MMP-9), α-smooth muscle actin (α-SMA), transforming growth factor-β1 (TGF-β1) and vascular endothelial growth factor (VEGF) levels [42, 45]. Also, the therapeutic effect of MSCs were shown on chemical injuries of the cornea using a range of delivery hydrogels such as nanofiber scaffold [46], amniotic membrane [43], and polysaccharide hydrogel [47].

Within the first part of the present study, we set out to investigate if the clinically relevant cell line, MAPC, can maintain viability and function following encapsulation within an alginate hydrogel and if the hypothermic storage (at 4 and 15°C) of these cells is possible without compromising their function and viability. Sodium alginate has been used to preserve MSCs in ambient and hypothermic temperatures where it was found that any small changes in temperature could affect cells viability and recovery [48, 49]. It was also shown that MSCs maintain their viability and function following storage and release from encapsulation [27]. Hence, we assessed MAPC viability and cell adhesion following storage and release from encapsulation, as they can be considered a therapeutically robust cell that could possibly assist directly in the repair of the cornea via release of paracrine factors alone. Indeed, our data support this hypothesis as the MAPC were shown to remain viable and functional following alginate encapsulation and hypothermic storage at both 4 and 15°C for 72 hours. Prominently, alginate was shown to have a key role in maintaining MAPC survival and function compared to non-encapsulated MAPC. Despite the absence of serum in encapsulation, cells showed high functional recovery and efficiency. Morphologically, MAPC are spindle-shaped fibroblastic-like cells, with large doubling potential and multilineage differentiation [48, 49]. In agreement with the literature [19, 28, 29], MAPC grown in culture showed similar morphology, importantly, there was no change in this morphology (after storage) indicating that the cells had not undergone any changes. This is supported by our previous work in which adipose-derived MSCs were shown not have remained undifferentiated following the same storage regime

Here we sought to demonstrate for the first time that alginate encapsulated MAPC, following hypothermic storage, maintain the ability to release soluble factors in response to the environmental cues when returned to normthermia. Thus, in the second part of our study, a simple scratch assay was employed to mimic a corneal stroma wound i*n vitro*. The data showed alginate encapsulated MAPC to have a significant effect on scratch closure rates over the first 30 hours. Moreover, this could only be attributed to the ability of encapsulated MAPC to release soluble factors from within the scaffold as the cells were unable to migrate out of the hydrogel. Interestingly the presence of alginate gel (control) in wound-healing cultures was shown to have attenuated the rate of wound healing and cell proliferation whilst increasing collagen production. Ion-exchange reaction happens when alginate is present in culture between the calcium ions in the alginate and the sodium ions in the medium [50, 51]. Upon reaching a certain degree of absorption of calcium, alginate starts to swell and dissolved partially, which could explain the effect of alginate control on the corneal stromal wound-healing.

Corneal stromal wound response involves a sequence of events that aim to fix and return the cornea to its normal structure and function. Upon corneal stromal injury, the normally inactive corneal stromal keratocytes differentiate to myofibroblasts to synthesize collagen and extracellular matrix components. The activated myofibroblasts express aSMA, vimentin and desmin to repopulate the wound via migration and proliferation [52, 53]. Growth factors are produced in this wound, and are well known to play an important role in wound healing i.e. TGF-ß, basic fibroblast growth factor (FGF-2) and platelet-derived growth factor (PDGF) [54]. TGF-ß effects the production of ECM components such as collagen, laminin and fibronectin, also it effects fibroblasts proliferation. Upon wound closure, myofibroblasts suppress their alpha SMA, vimentin and desmin expression [52, 55]. In our system, it is unknown if encapsulated MAPC produced TGF-ß to activate the keratocytes to myfibroblasts to enhance their migration and proliferation. However, as it was shown that MAPC have a favorable effect on the scratch-closure in a non-contact system, this indicates that MAPC do produce soluble factors compared to the controls. We suggest that these growth factors may include hepatocyte growth factor (HGF) and keratinocyte growth factor (KGF) that are known to be important in corneal stromal wound healing [53].

Collagen is produced by fibroblastic cells in response to wounding. Following *in vivo* corneal stromal injury, collagen type I and III deposition increases at the wound site, depending on the wound type this deposition could be delayed [56, 57]. This could explain collagen disposition we see experimentally within our simple scratch assay. The data shown suggests that cells produce less collagen when they are migrating (as shown in Fig 4). This resulted in low collagen expression per cell in serum containing media (as cells were highly proliferative) and high collagen expression per cell in alginate alone (where cells did not proliferate), indeed it has been suggested that calcium alginate improves collagen production in wound closure [58, 59]. However, in the presence of MAPC and, the data show a combination of migration and increased collagen expression, which although not shown here as significant could still prove useful in wound healing.

Although these results provide compelling evidence that MAPC encapsulated in alginate, stored at hypothermic temperatures, and applied to human corneal stromal cells enhance the rate of healing, the underpinning paracrine mechanism remains unclear. As mentioned before, MAPC have similarities to MSCs, and it is known that MSCs do express TGF-β, HGF, FGF, and TSG-6, which in turn can affect cell proliferation and migration, while improving corneal healing [60, 61]. Thus, we would expect these factors to be also released from MAPC in response to corneal wound.

## Conclusion

Alginate encapsulation significantly improved functional cell recovery following storage at both 4 and 15°C. Alginate encapsulated MAPC enhance the rate of wound closure in human corneal stromal cells *in vitro*. The wound healing effects of encapsulated MAPC is enhanced following hypothermic storage. Overall, the work lends considerable evidence to the novel concept of a storable cell-laden gel with the caveat that this needs to be investigate within an *in vivo* setting to confirm our hypothesis that alginate encapsulated MAPC have the ability to improve wound healing and consequently reduce corneal scar formation.
